# Postoperative Gabapentin to Prevent Postoperative Pain: A Randomized Clinical Trial

**DOI:** 10.5812/aapm.4744

**Published:** 2012-09-13

**Authors:** Mahdi Panah Khahi, Shaqayeq Marashi, Mohammad Reza Khajavi, Atabak Najafi, Amirabbas Yaghooti, Farsad Imani

**Affiliations:** 1 Department of Anesthesiology, Faculty of Medicine, Iran University of Medical Sciences, Tehran, IR Iran

**Keywords:** Gabapentin, Pain, Analgesia

## Abstract

**Background:**

Gabapentin is an anticonvulsant that has postoperative analgesic effects but there are limited studies on its postoperative administration.

**Objectives:**

The present study was conducted to evaluate the effect of the postoperative oral gabapentin on pain and morphine consumption.

**Patients and Methods:**

In a double blind, randomized study, 64 patients undergoing internal fixation of tibia under spinal anesthesia were randomly assigned to receive oral gabapentin or placebo immediately after the surgery. Pain scores were recorded at time points of 2, 12 and 24 hours postoperatively using visual analog scale (VAS). Time duration from the end of surgery until morphine administration and total morphine requirement in the first 24 hours were recorded.

**Results:**

The estimated duration of surgeries was 120-150 minutes. VAS score was not significantly different between the two groups at 2, 12 and 24 hours after surgery. There was no significant morphine consumption difference between the groups.

**Conclusions:**

Our study showed no significant analgesic efficacy of oral gabapentin 300 mg immediately after tibia internal fixation surgery under spinal anesthesia at time points of 2, 12 and 24 hours postoperatively.

## 1. Background

Nowadays, a variety of new drugs, devices, analgesic techniques and preventive approaches are available for anesthesiologists including patient-controlled analgesia, multimodal analgesia and pre-emptive analgesia (1-4). Gabapentin has an analgesic (5) and opioid-sparing effect (1, 4, 6) both at rest and with movement (7) in acute postoperative pain management (1, 8-13). Postoperative pain is not purely nociceptive and may have other (inflammatory and visceral) components (14); so using drugs that act on different analgesic mechanisms seems logical (15-18). Gabapentin has proved an anti-inflammatory effect in animal model (19, 20). In human, Werner’s study showed gabapentin reduces primary mechanical allodynia in acute inflammation following a thermal injury (21). Dirks’ study linked data from animal models and clinical trials for chronic pain by investigating the effect of gabapentin on acute nociception and experimentally induced cutaneous hyperalgesia in healthy volunteers and indicated that gabapentin profoundly suppressed established cutaneous sensitization (22). Gabapentin appears to be an acceptable alternative for cyclooxygenase-2 inhibitors for perioperative pain management and short-term use as an adjuvant to opioid analgesics (23). Based on a systematic review, perioperative oral gabapentin is a useful adjunct for the management of postoperative pain that provides analgesia through a different mechanism from opioids and other analgesic agents and would make a reasonable addition to a multimodal analgesic treatment plan (24). In recent years, many studies have been conducted on pre-incisional or pre and post-incisional administration of gabapentin (25) and its efficacy has almost been proved but there are limited studies on its post-incisional efficacy alone (effect on secondary phase of injury). Clinical trials to evaluate post incision gabapentin are lacking and more studies on dosing and duration of treatment with gabapentin would be needed.

## 2. Objectives

The present study was designed to evaluate the effect of the postoperative oral gabapentin on pain and morphine consumption amount of patients after internal fixations of tibia under spinal anesthesia.

## 3. Patients and Methods

The medical university ethics committee approved this study and a written informed consent was obtained from each patient. In this double blind, randomized controlled clinical trial conducted in a specialized and subspecialized university hospital from 31.08.2009 until 31.08.2010, 64 patients with ASA physical status of 1 or 2, aged 16-70 years who were scheduled for internal fixations of tibia were recruited.

Patients with known sensitivity to gabapentin, history of seizure, positive history of gabapentin consumption, psychiatric disorders, drug abuse, known liver or renal disease, chronic pain syndromes, and the patients who had an intake of analgesic drugs 24 hours before the test were excluded from the study.

After the placement of standard monitoring [ECG, heart rate, pulse oximetry and non-invasive blood pressure (NIBP)], a midline lumbar puncture was performed with a 24 gauge pencil point needle at the level of L3-L4 while the patient was held in sitting position and 3 mL (15 mg) of isobaric bupivacaine (0.5%) was injected for all patients and they were turned to supine position. The surgeon was allowed to start the operation after complete sensory motor blockade at the level of T8-T10. The patients received 1-2 mg midazolam.

The patients were randomly assigned into two groups of 32 by a table of random numbers. Patients in the gabapentin group, received 300 mg gabapentin orally immediately after being taken to recovery room and were eligible to drink water with capsule. The placebo group received a placebo capsule at the same time.

In the preoperative visit, the participants were trained how to use a visual analogue scale (VAS range 0-10) after the surgery. Patients’ pain scores were recorded by another anesthetist unaware of patients’ group at 2-12 and 24 hours after surgery on a visual analogue scale. time from the end of surgery until the first injection of morphine administeredon demand was recorded. Total amount of morphine administration by the responsible physician of the ward on the first day was recorded, too.

The Statistical Package for Social Sciences version 10.0 (SPSS Inc. Chicago, IL, USA) was employed to analyze data. Kolmogorov-smirnov z test was performed on the data to assess distribution normality. The analyses were performed based on chi square, independent t tests and mann-whitney U test. The summary of the data is presented as mean ± SD and the statistical significance was reported when P value was < 0.05.

## 4. Results

The estimated duration of surgeries was 120-150 minutes. All the patients had the complete sensory motor blockade at the level of T8-T 10 before the beginning of surgery. Patients were pain free, so analgesics was not administered.

There was no difference in age and sex between the two groups (Table 1). VAS score was not significantly different between the two groups at 2, 12 and 24 hours after surgery (Table 2).

**Table 1:** Demographic Data

	Gabapentin	Placebo	*P* value
Age, y, Mean ± SD	32.81 ± 2.33	32.25 ± 2.24	0.856
Sex, male/female ratio	24/8	23/9	0.499
ASA, I/II ratio	22/10	18/14	0.179
Duration to 1^st^ dose of MS, h, Mean ± SD	3.03 ± 0.71	3.28 ± 0.59	0.517
Required dose of MS, mg, Mean ± SD	6.25 ± 0.62	5.72 ± 0.79	0.577

Abbreviations: MS, morphine sulfate; ASA, American Society of Anesthesiology.

**Table 2:** VAS Score Between Groups Based on Time

	2 Hour	12 Hour	24 Hour
Gabapentin, Mean ± SD	5.34 ± 0.79	6.97 ± 0.73	2.69 ± 0.54
Placebo, Mean ± SD	5.16 ± 0.98	7.44 ± 0.87	2.47 ± 0.34
*P* value	0.76	0.406	0.457

Total morphine requirement on the 1st day after surgery and the time to administer the first dose of morphine on demand was almost similar in gabapentin and placebo groups (Table 1). No special side effect was observed in the gabapentin group.

## 5. Discussion

The current study indicated no significant analgesia efficacy of 300 mg oral gabapentin when it was consumed immediately after surgery of internal fixation of tibia under spinal anesthesia. The total amount of morphine requirement on the 1st day in the gabapentin group was not different from the placebo. In a study by Pandey, both pre and post incision administration of gabapentin were equally effective in reducing postoperative pain in subjects undergoing open donor nephrectomy and both groups also consumed less fentanyl (26). There are 4 methodological differences between Pandey`s and the current study:

1^st^, The dose of gabapentin was 600 mg but in the current study, it was 300 mg.

2^nd^, The time points at which pain scores were recorded. While gabapentin peak concentration is after 2-3 hours (22) and its half-life is 5-9 hours, (25, 27) in the current study, patients’ pain scores at 2-12-24 hours after surgery were recorded and in Pandey`s study, the times were 0-6-12-18-24.

3^rd^, The time interval between the incision and taking the medication. In this study, post incision gabapentin was administered via nasogastric tube intraoperatively while in our study, gabapentin administration was postoperative.

4^th^, In the current study type of anesthesia was spinal versus general anesthesia in Pandey`s. Therefore, the ineffectiveness of gabapentin in our study may be due to the delay in its administration and also the altered central processing.

In a study by Hussain Khan, pre and post-incision gabapentin administration was found effective in pain management (28). In this study, doses of gabapentin were 600, 900 or 1200 mg which are twice more than that of the current study (Larger doses can produce adverse effects which is a concern). In conclusion, more studies on dosing, timing and postoperative administration of gabapentin seem promising.
